# Morphological characteristics of high myopes complicated by serous retinal detachment with dome-shaped macula or inferior staphyloma

**DOI:** 10.1007/s00417-025-06802-z

**Published:** 2025-03-25

**Authors:** Jeong Hyun Lee, Woosung Jeon, Min Seok Kim, Kwangsic Joo, Se Joon Woo, Joo Young Shin, Jeeyun Ahn

**Affiliations:** 1 Department of Ophthalmology, Seoul National University College of Medicine, Seoul, Korea; 2 Department of Ophthalmology, Seoul Metropolitan Government-Seoul National University Boramae Medical Center, Seoul, Korea; 3 Department of Ophthalmology, Seoul National University Hospital, Seoul, Korea; 4 Department of Ophthalmology, Seoul National University Bundang Hospital, Seongnam, Korea

**Keywords:** Dome-shaped macula, Inferior staphyloma, Serous retinal detachment, High myopia

## Abstract

**Purpose:**

To investigate the morphological characteristics of dome-shaped macula (DSM) and inferior staphyloma complicated by serous retinal detachment (SRD).

**Methods:**

Electronic medical records and multimodal images of patients diagnosed with DSM and inferior staphyloma were retrospectively reviewed. Morphological features, including axial length, curvature height, orientation of the dome, presence of retinal pigment epithelial detachment, choroidal vascular features such as subfoveal choroidal thickness, variations in choroidal thickness, presence of abrupt changes in choroidal thickness and presence of large choroidal vessels were analyzed.

**Results:**

Fifty-three eyes of 37 patients were included, of which 33 eyes had DSM and 20 eyes had inferior staphyloma, and 15 (28.3%) were complicated with SRD. Four (12.1%) of the DSM and 11 (55.0%) of the inferior staphyloma eyes had SRD. On univariate analysis of risk factors for SRD, shorter axial length (*p* = 0.002), presence of inferior staphyloma (*p* = 0.004), higher subfoveal curve height (*p* = 0.009), thicker subfoveal choroidal thickness (*p* = 0.038), greater variation in choroidal thickness (*p* = 0.005), presence of both abrupt changes in choroidal thickness (*p* < 0.001) and large choroidal vessels (*p* = 0.001) showed a significantly higher risk. On multivariate analysis, shorter axial length (*p* = 0.038) and presence of abrupt changes in choroidal thickness (*p* = 0.008) were identified as significant risk factors for SRD.

**Conclusion:**

SRD was more prevalent in eyes with inferior staphyloma compared to DSM. Shorter axial length and abrupt changes in choroidal thickness were associated risk factors for SRD.

**Supplementary Information:**

The online version contains supplementary material available at 10.1007/s00417-025-06802-z.

## Abstract (highlights)

**Key messages:**

*****What is known***:**

- Dome-shaped macula (DSM) and inferior staphyloma have a unique feature in that the abrupt change in eyeball curvature occurs within the macular area.
- DSM and inferior staphyloma can be associated with serous retinal detachment (SRD) in the macular area, and the pathophysiology is unknown.

*****What is new***:**

- Shorter axial length and abrupt change of choroidal thickness are associated risk factor for SRD in eyes with DSM and inferior staphyloma.
- Abrupt change of choroidal thickness and SRD were more prevalent in inferior staphyloma than in DSM.

**Supplementary Information:**

The online version contains supplementary material available at 10.1007/s00417-025-06802-z.

## Introduction

With the introduction of optical coherence tomography (OCT) in daily practice, various features of the macular in highly myopic eyes have been described, with some newly identified as the cause of visual decline [1, 2]. As technology in imaging the posterior segment of the eye advances, various other features are continuously being identified and described. Despite ongoing efforts to classify myopic maculopathy, a unified grading system encompassing the wide spectrum of morphologic features is still lacking [3].

Dome-shaped macula (DSM), first described in 2008 by Gaucher et al., was defined as an inward bulge within the chorioretinal posterior concavity in the macular area of highly myopic eyes with type I or II posterior staphyloma [4]. Detection of DSM became possible with the advent of OCT, as the macular bulge was difficult to detect with fundus biomicroscopy. The prevalence of DSM in highly myopic eyes is reported to be 9.3 to 10.7% [4, 5]. In the first report by Gaucher et al., 10 out of 15 eyes showed a shallow serous retinal detachment (SRD) on the top of the DSM. Subsequent articles reported a highly variable incidence of SRD in DSM, ranging between 1.8 and 66.7% [4–7].

Inferior staphyloma, previously classified as a type V staphyloma, is a type of primary posterior staphyloma associated with myopia [8]. When the superior edge of the staphyloma transverses the macula, complications such as choroidal neovascularization, polypoidal choroidal vasculopathy, or SRD may ensue, resulting in visual deterioration [9]. Inferior staphyloma is sometimes accompanied by tilted disc syndrome, peripapillary crescent, myopia, and RPE thinning at the inferonasal fundus [10]. It can also be accompanied by SRD at its superior border, usually the macula. Nakanishi et al. reported SRD at the border of the inferior staphyloma in 13 out of 32 eyes (41%) with no associated polypoidal choroidal vasculopathy or choroidal neovascularization [11].

Both DSM and inferior staphyloma share common features. According to the orientation of the protrusion, three anatomical types of DSM have been described; vertically oriented dome, horizontally oriented dome, and round-shaped dome. Inferior staphyloma can be thought of as a horizontally oriented dome, with the inferior protrusion having a steeper curvature than the superior protrusion. Abrupt changes in curvature and SRD are found in the macular region in these highly myopic patients, and the cause of the SRD and whether treatment is necessary or effective remains to be determined [12, 13].

Herein, we investigated the morphological characteristics of DSM and inferior staphyloma complicated by SRD in high myopic eyes to further elucidate the underlying pathophysiology.

## Methods

### Study design and participants

**Fig. 1:**
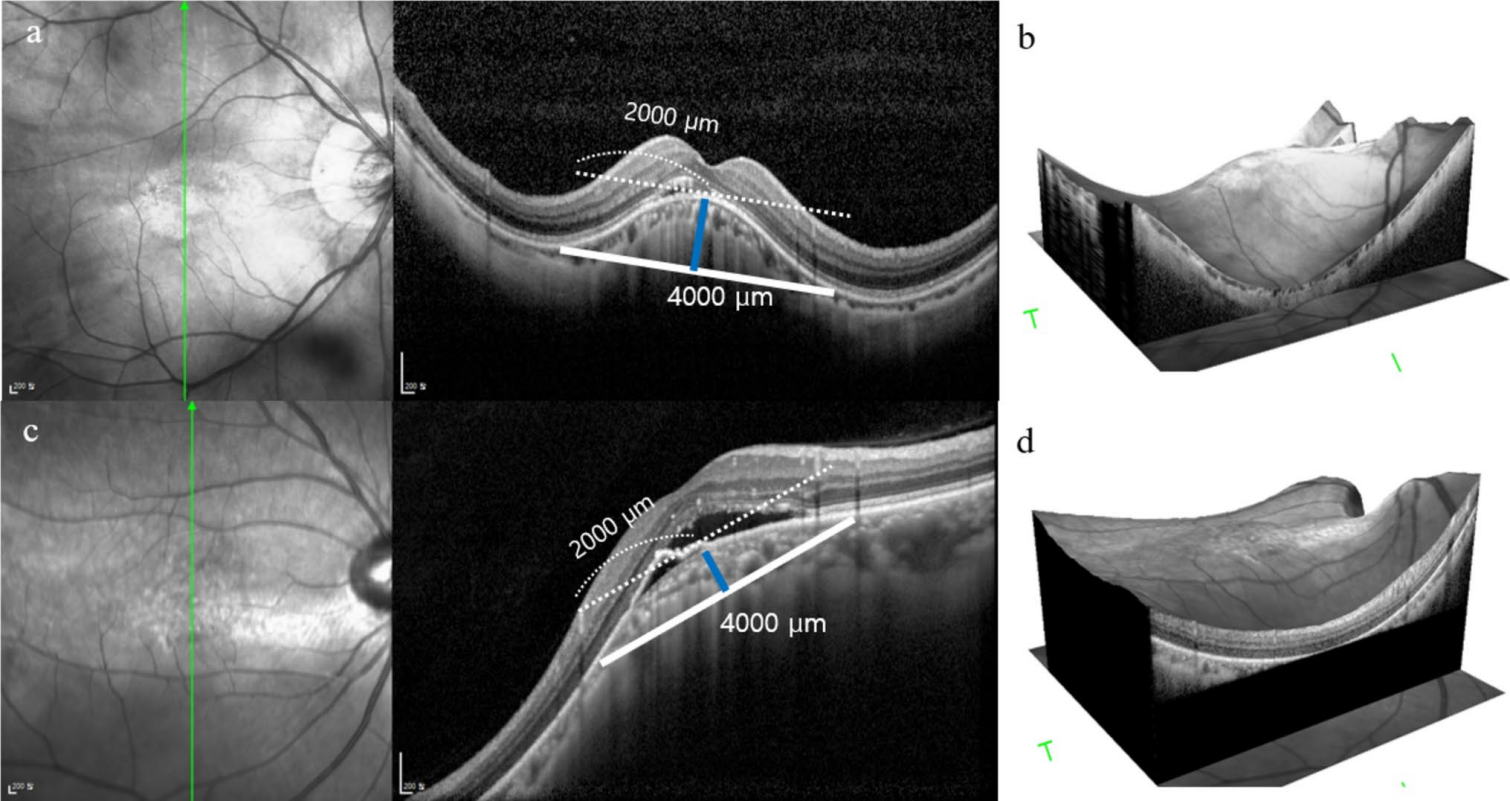
Optical coherence tomography images demonstrating the measurement of subfoveal curve height (blue line) in an eye with dome-shaped macula **a**, **b** and inferior staphyloma **c**, **d** complicated by serous retinal detachment. A virtual 4000 µm line centered on the fovea and parallel to the line tangential to the curve apex was drawn and subfoveal curve height was defined as the shortest vertical distance between the virtual line and the curve apex along the outer border of retinal pigment epithelium. Three-dimensional reconstruction images are shown in **b** and **d**

A multicenter, retrospective, cross-sectional study was conducted, reviewing the electronic medical records of highly myopic patients diagnosed with DSM and inferior staphyloma with or without SRD from January 1st, 2012 to September 31st, 2022 at two referral hospitals: Seoul Metropolitan Government-Seoul National University Boramae Medical Center and Seoul National University Bundang Hospital. This study complies with the Declaration of Helsinki and was approved by the Institutional Review Boards at both hospitals (No. 30–2023-64 and B-2405–899-401). High myopia was defined as a refractive error ≤ >−6 diopters (D) or an axial length ≥ 26 mm. DSM was diagnosed when presence of an inward bulge of the macular RPE of > 50 µm in the vertical or horizontal OCT scans was found (Fig. 1). Inferior staphyloma was diagnosed on multimodal imaging, including an OCT vertical scan, where an abrupt change of curvature is found at the macula to form a staphyloma at the inferior globe (Fig. 1). Exclusion criteria included the presence of choroidal neovascularization, any other ocular comorbidity such as age-related macular degeneration, diabetic retinopathy, glaucoma, retinal vascular diseases, medial opacity significant enough to affect OCT image quality such as corneal opacity or dense cataract, and a history of intraocular surgery or intravitreal injection.

### Data collection and image analysis

Baseline demographics and ocular examinations were collected. Patients underwent a complete ocular examination, including automated refraction test, best-corrected visual acuity (BCVA) measurement, tonometry, slit-lamp biomicroscopy, and indirect fundus examination. Axial length was measured using IOL Master 500 (Carl Zeiss Meditec, Jena, Germany) or IOL Master 700 (Carl Zeiss Meditec, Jena, Germany). Fundus photography was taken with wide-angle fundus imaging using California Optos (Optos, Marlborough, MA, USA), and spectral-domain OCT (Spectralis; Heidelberg Engineering, Heidelberg, Germany) to evaluate the presence of DSM/inferior staphyloma. Using enhanced depth imaging (EDI) mode, macular volume scans and single vertical and horizontal scans encompassing the macula were obtained. The 768 × 496 macular volume scan covered a 30° × 25° field that was a 9.6 × 8.0 mm area centered on the fovea. Total of 31 cross-sectional B-scan images were obtained, each composed of 512 A-scans. ART was set at 100. The presence of inferior staphyloma and DSM was determined using multimodal imaging, including three-dimensional reconstruction of the macular volume scan (Fig. 1B and D).

**Fig. 2:**
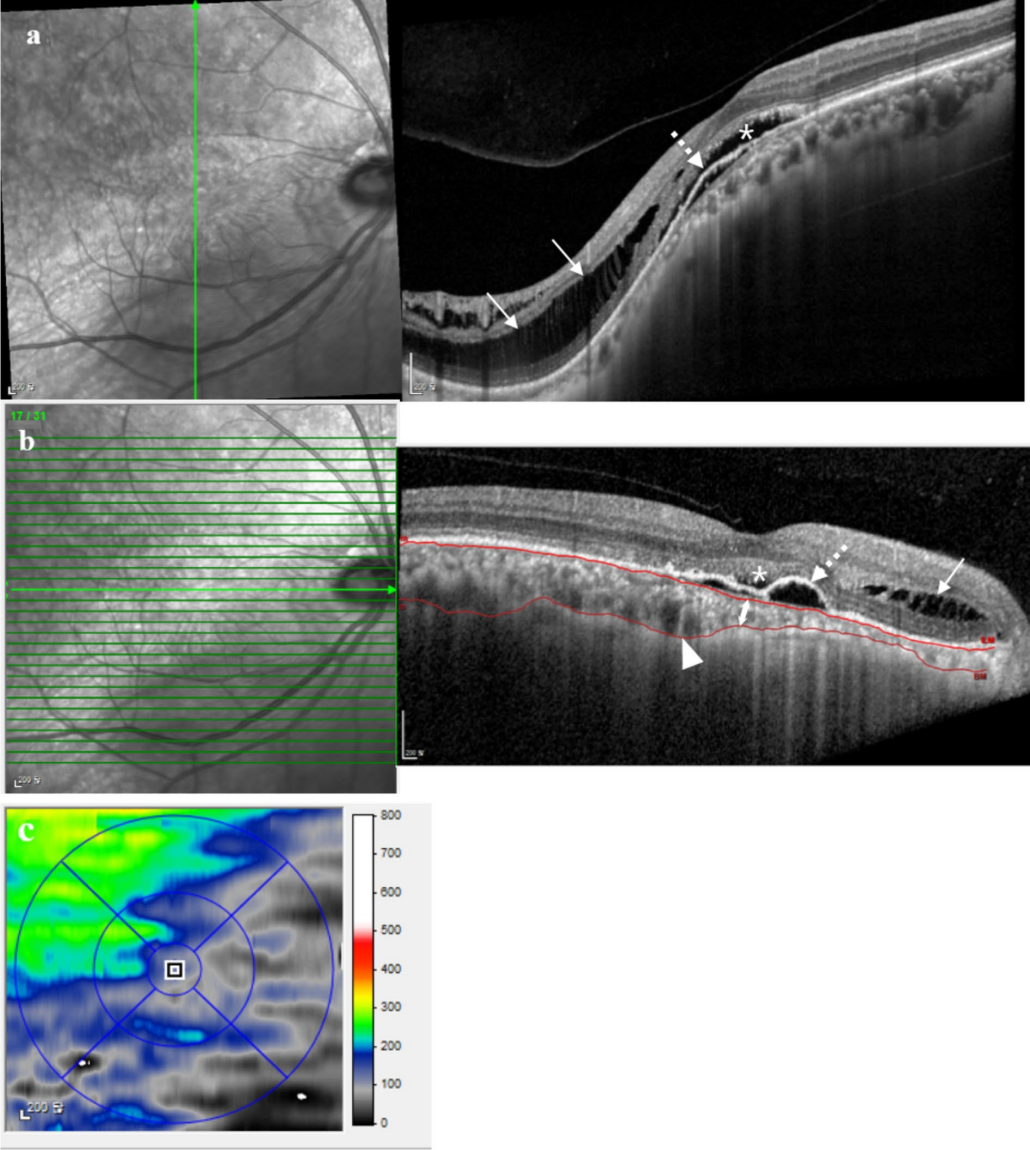
Single vertical scan **a**, macular volume scan **b**, and choroidal thickness topographic map **c** of an inferior staphyloma complicated by serous retinal detachment (asterisk), pigment epithelial detachment (dashed arrow), and extrafoveal retinoschisis (arrow) are shown. Subfoveal choroidal thickness (double arrow), presence of a large choroidal vessel (arrowhead) and presence of abrupt changes in choroidal thickness are demonstrated. Note the more than 25% change in choroidal thickness within 1500 µm in the superotemporal parafoveal region on **c**

**Fig. 3:**
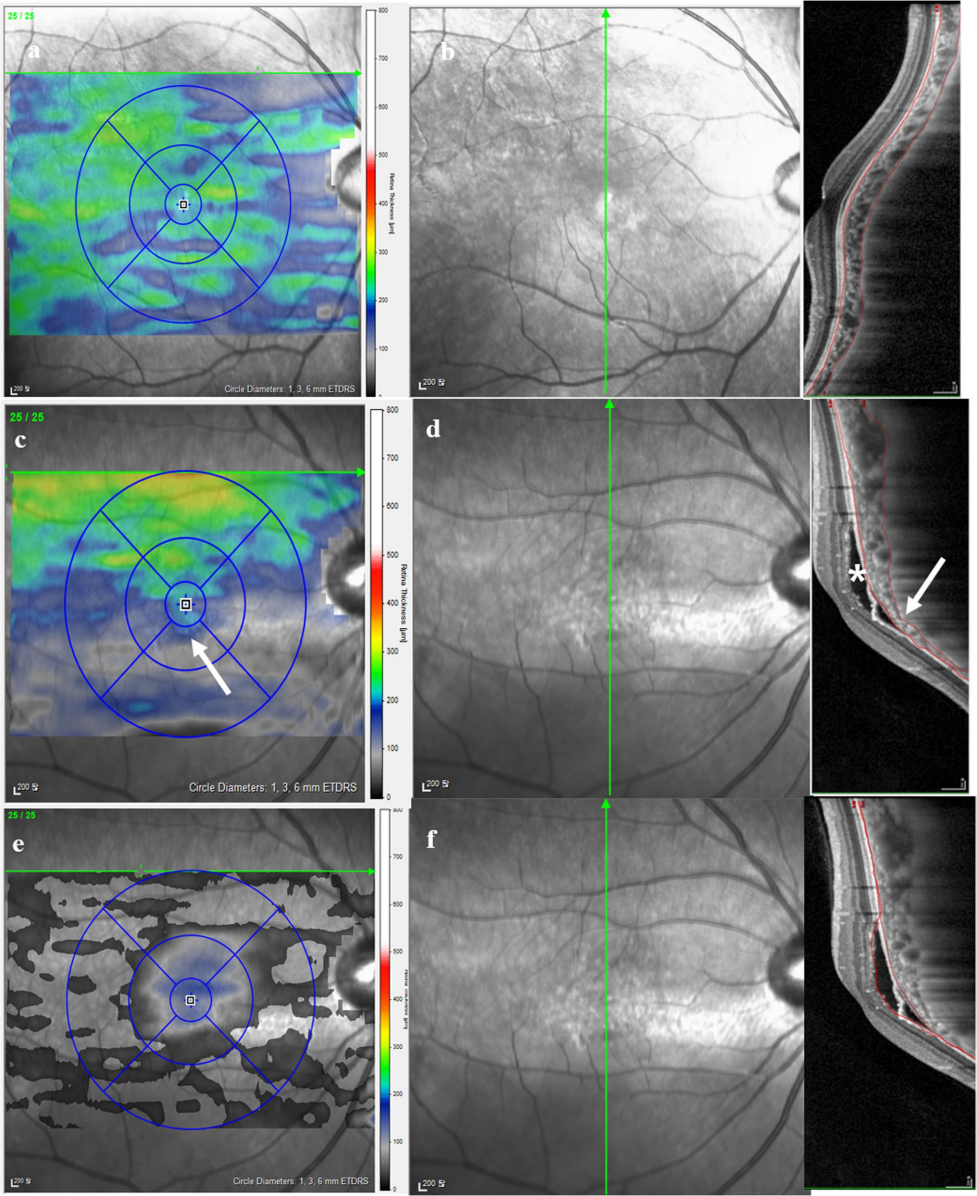
Representative images of dome-shaped macula without serous retinal detachment **a**, **b** and inferior staphyloma **c**, **d** with serous retinal detachment. Choroidal thickness topographic maps are shown in **a** and **c**. **e** is a topographic map illustrating the localized area of serous retinal detachment, created by manually adjusting the auto-segmentation lines to align with the borders of the detachment, as depicted in **f**. Notably, the area of abrupt choroidal thickness change (indicated by the arrow) is situated near the region of serous retinal detachment (indicated by the asterisk) within the inferior staphyloma **c**, **e**

Based on the macular volume scan and either horizontal or vertical scans that show steeper curvature, morphological analyses including orientation of the dome, subfoveal curve height measurement, presence of SRD, and presence of ocular comorbidity other than SRD (pigment epithelial detachment [PED], cystoid macular edema [CME], retinoschisis, macular hole, epiretinal membrane [ERM]) were conducted. Subfoveal curve height was measured as shown in Fig. 1, by drawing a virtual 4000 µm line centered on the fovea and parallel to the line tangential to the curve apex. Subfoveal curve height was defined as the shortest vertical distance between the virtual line and the curve apex along the RPE outer border. Choroidal features assessed included subfoveal choroidal thickness, variations in choroidal thickness, presence of abrupt changes in choroidal thickness and presence of large choroidal vessels (Fig. 2). Choroidal thickness was measured from the outer border of the hyperreflective line corresponding to the RPE to the hyporeflective line corresponding to the sclerochoroidal interface. Variations in choroidal thickness was determined by the difference between the thickest and thinnest region of the choroid. A topographic map showing choroidal thickness was generated by manually adjusting the auto-segmentation lines on volume scans to cover the choroidal thickness (Fig. 2B and C) using Heidelberg Eye Explorer software (version 6.9) (Fig. 3). The presence of abrupt changes in choroidal thickness was defined as a choroidal thickness change of at least 25% within a distance of 1500 µm along the length of the respective scan, based on previous studies that found maximum difference between the mean subfoveal choroidal thickness and the thinnest choroidal thickness of normal adults within the 3 mm ETDRS circle to be ≤ 20% [14, 15]. The presence of abnormal large choroidal vessels was defined as choroidal vessels > 200 µm in diameter, based on a previous article measuring the mean value of the three largest choroidal vessels in normal adults within the 3 mm ETDRS circle to be 122.5 ± 20.7 µm [16]. Measurement of subfoveal curve height, subfoveal choroidal thickness, and variation of choroidal thickness was independently performed by two investigators (J.H.L. and J.Y.S.), and the average of their measurements was recorded to ensure consistency and reduce individual bias. The presence of abrupt change in choroidal thickness and large choroidal vessel was also independently assessed by the same two investigators. In cases of disagreement, a third investigator (J. A.) reviewed the images, and the final decision was made based on the majority rule. A high level of agreement between the two primary investigators further supports the reliability of these measurements.

### Statistical analysis

Statistical analyses were performed using IBM SPSS statistics ver. 27.0 (IBM Corp, Armonk, NY, USA). Numerical data were expressed as mean and standard deviation, and categorical variables as absolute frequency and percentage. Statistical comparisons between patients with or without SRD was performed with the Mann–Whitney test for numerical variables and Fisher’s exact test for categorical variables. Since data from both eyes of some patients were included in the analysis, adjustments for multiple comparisons were applied to account for inter-eye correlation. This adjustment was also applied in the univariate and multivariate logistic regression analyses of factors associated with SRD. Univariate and multivariate logistic regression analysis of factors associated with SRD were also performed. A *P* value of < 0.05 was considered statistically significant.

## Results

A total of 53 eyes of 37 patients were included, and baseline demographics are shown in Table 1. Thirty-three eyes had DSM and 20 eyes had inferior staphyloma. Of the 33 eyes with DSM, 30 were horizontally oriented, 3 were round, and none were vertically oriented. SRD was found in 15 eyes (28.3%), of which 4 eyes were DSM, and 11 eyes were inferior staphyloma. All SRD were found either on top of the dome or at the edge of the inferior staphyloma, both under the fovea. Presence of abrupt change in choroidal thickness and large choroidal vessels were both found in 15 eyes (28.3%). Among complications other than SRD, ERM (9 eyes, 17.0%) was the most common, and macular hole (1 eye, 1.9%) was the least common. Retinoschisis (7 eyes, 13.2%) was found only in the extrafoveal region.

**Table 1:** Baseline demographics

	*N* = 53
Age (years)	57.23 ± 16.07
Sex (male [%]/female [%])	13 (24.5)/40 (75.5)
Best-corrected visual acuity (logMAR)	0.35 ± 0.32
Tonometer (mmHg)	12.19 ± 3.53
Spherical equivalent (diopters)	- 4.69 ± 6.65
Lens status (phakia [%]/pseudophakia [%])	36 (67.9)/17 (32.1)
Axial length (mm)	28.29 ± 3.69
Presence of serous retinal detachment (n [%])	15 (28.3)
Dome-shaped macula (n [%])/Inferior staphyloma (n [%])	33 (62.3)/20 (37.7)
Subfoveal curve height (µm)	266.71 ± 153.83
Subfoveal ChT (µm)	119.41 ± 97.17
Variation of ChT (µm)	69.41 ± 65.64
Presence of abrupt changes in ChT (*n* [%])	15 (28.3)
Presence of large choroidal vessels (*n* [%])	15 (28.3)
Presence of comorbidities (*n* [%])	
Pigment epithelial detachment	4 (7.5)
Cystoid macular edema	4 (7.5)
Extrafoveal retinoschisis	7 (13.2)
Foveal retinoschisis	0 (0)
Macular hole	1 (1.9)
Epiretinal membrane	9 (17.0)

*N* number, *logMAR* logarithm of minimal angle resolution, *ChT* choroidal thickness

The comparison of eyes with or without SRD is listed in Table 2. Inferior staphyloma was more common than DSM in eyes with SRD (73.3% vs 26.7%, *p* = 0.002). Eyes with SRD had a higher subfoveal curve height (373.21 ± 80.44 µm vs 223.68 ± 156.80 µm, *p* < 0.001), thicker subfoveal choroidal thickness (204.93 ± 106.83 µm vs 87.05 ± 71.22 µm, *p* = 0.015), and greater variation in choroidal thickness (123.21 ± 82.58 µm vs 49.05 ± 44.38 µm, *p* = 0.001). The presence of abrupt changes in choroidal thickness (73.3% vs 10.5%, *p* < 0.001) and large choroidal vessels (66.7% vs 13.2%, *p* < 0.001) were more frequently found in eyes with SRD. Abrupt changes in choroidal thickness were most commonly located within the SRD (60%), followed by the edge (26.7%), and least commonly found away from SRD (13.3%). Large choroidal vessels were also most commonly located within the SRD (66.7%), followed by the edge (20%), and least commonly found away from SRD (13.3%). Among complications other than SRD, none of them was associated with presence of SRD. Axial length was shorter in eyes with SRD (25.55 ± 1.27 vs 29.67 ± 3.64 µm, *p* = 0.007).

**Table 2:** Comparison of eyes with and without serous retinal detachment

	SRD ( +) (*n* = 15)	SRD (-) (*n* = 38)	*P* value
Age (years)	55.07 ± 10.73	58.08 ± 17.78	0.268^1^
Sex (male [%])/female [%])	3 (20.0)/12 (80.0)	10 (26.3)/28 (73.7)	0.461^2^
Best-corrected visual acuity (logMAR)	0.34 ± 0.23	0.35 ± 0.35	0.652^3^
Tonometer (mmHg)	11.87 ± 3.60	12.32 ± 3.54	0.995^3^
Spherical equivalent of refraction (diopters)	- 4.41 ± 3.14	– 4.80 ± 7.67	0.948^3^
Lens status (phakia [%] /pseudophakia [%])	13(86.7)/2 (13.3)	22 (57.9)/16 (42.1)	0.061^4^
Axial length (mm)	25.55 ± 1.27	29.67 ± 3.64	0.007^3^
Dome-shaped macula (n [%])/Inferior staphyloma (n [%])	4 (26.7)/11 (73.3)	29 (76.3)/9 (23.7)	0.002^3^
Subfoveal curve height	373.21 ± 80.44	223.68 ± 156.80	< 0.001^3^
Subfoveal ChT (µm)	204.93 ± 106.83	87.05 ± 71.22	0.015^3^
Variation of ChT (µm)	123.21 ± 82.58	49.05 ± 44.38	0.001^3^
Presence of abrupt change in ChT (n [%])	11 (73.3)	4 (10.5)	< 0.001^3^
Presence of large choroidal vessels (n [%])	10 (66.7)	5 (13.2)	< 0.001^3^
Presence of comorbidities (n [%])			
Pigment epithelial detachment	4 (26.7)	0 (0)	0.103^4^
Cystoid macular edema	2 (13.3)	2 (5.3)	0.440^3^
Extrafoveal retinoschisis	2 (13.3)	5(13.2)	0.989^3^
Foveal retinoschisis	0 (0)	0 (0)	
Macular hole	0 (0)	1 (2.6)	1.000^4^
Epiretinal membrane	0 (0)	9 (23.7)	0.508^2^

*N* number, *logMAR* logarithm of minimal angle resolution, *SRD* serous retinal detachment, *ChT* choroidal thickness ^1^Mann-Whitney test ^2^Fisher’s exact test ^3^Linear Mixed Models ^4^Generalized Linear Mixed Models

On logistic regression analysis, shorter axial length, presence of inferior staphyloma, higher subfoveal curve height, thicker subfoveal choroidal thickness, greater variation in choroidal thickness, presence of abrupt changes in choroidal thickness, and presence of large choroidal vessels were significantly associated with a higher risk of SRD (*p* < 0.05), as listed on Table 3. On multivariate analysis, only shorter axial length (*p* = 0.038) and presence of abrupt changes in choroidal thickness (*p* = 0.008) remained significant risk factors for SRD.

**Table 3:** Univariate and multivariate logistic regression analyses using a mixed-effects model to account for inter-eye correlation in risk factors associated with serous retinal detachment in eyes with dome-shaped macula and inferior staphyloma

	Univariate analysis			Multivariate analysis		
Clinical variables	Odds Ratio	95% Confidence Interval	*P* value	Odd Ratio	95% Confidence Interval	*P* value
Age	1.014	0.972–1.057	0.511			
Male	0.541	0.074–3.974	0.539			
Best-corrected visual acuity	2.203	0.216–22.506	0.498			
Tonometer	1.042	0.837–1.298	0.704			
Spherical equivalent of refraction	1.007	0.893–1.135	0.911			
Pseudophakia	0.220	0.039–1.246	0.086			
Axial length	1.854	1.274–2.699	0.002	1.631	1.030–2.584	0.038
Inferior staphyloma	8.698	2.051–36.892	0.004	0.368	0.023–6.019	0.475
Subfoveal curve height (µm)	1.007	1.002–1.012	0.009	1.005	0.998–1.013	0.165
Subfoveal ChT (µm)	1.0152	1.008–1.316	0.038	1.004	0.987–1.021	0.654
Variation of ChT (µm)	1.019	1.006–1.033	0.005	1.003	0.980–1.026	0.818
Presence of abrupt changes in ChT (*n* [%])	22.264	4.594–107.8892	< 0.001	11.529	1.945–68.344	0.008
Presence of large choroidal vessels (*n* [%])	12.795	2.864–57.155	0.001	1.414	0.081–24.571	0.808

*N* number, *logMAR* logarithm of minimal angle resolution, *ChT* choroidal thickness

Of the 15 eyes with SRD, 12 eyes received intravitreal injection of an anti-vascular endothelial growth factor (anti-VEGF) agent at least once, and the treatment outcomes are summarized in Table 4. Most eyes were started on bevacizumab treatment (10 eyes), while 2 eyes were initially treated with aflibercept. All eyes received consecutive injections except for 2 cases. There were no significant changes in BCVA between baseline and 1 month after the last injection (*p* = 0.336), and also between 1 month after the last injection and the last visit (*p* = 0.345). SRD was unresponsive to treatment (stationary or increased) in 8 eyes, while SRD decreased but did not completely regress in 3 eyes, and completely regressed in 1 eye. Over the follow-up period of 44.75 ± 40.20 months without any additional treatment, SRD decreased in 4 eyes and increased in 4 eyes. Representative OCT images of patients with DSM and inferior staphyloma who showed response after intravitreal injection are shown in Supplementary Fig. 1.

**Table 4:** Treatment summary of eyes with serous retinal detachment

Eye	Sex	Age (yrs)	DSM/Inferior staphyloma	BCVA before injection (logMAR)	Number of injections	Type of anti-VEGF agent	BCVA 1 month after last injection (logMAR)	SRD status 1 month after last injection	BCVA at last visit (logMAR)	SRD status At last visit, compared to 1 month after last injection	Follow-up period (months)
1	F	56	inferior staphyloma	0.52	8	bevacizumab × 6, aflibercept × 2	0.52	increased	0.52	decreased but remaining	85
2	M	59	inferior staphyloma	0.30	2	bevacizumab × 2	0.30	stationary	0.22	stationary	17
3	F	76	inferior staphyloma	1.00	2	bevacizumab × 2	0.70	decreased but remaining	1.10	increased	58
4	F	47	DSM	0.05	2	bevacizumab × 2	0.05	stationary	0.22	increased	26
5	F	37	DSM	0.70	3	bevacizumab × 3	0.52	stationary	0.70	regressed	27
6	F	50	inferior staphyloma	1.00	2	bevacizumab × 2	0.82	stationary	1.70	regressed	79
7	F	44	DSM	0.30	2	bevacizumab × 2	0.40	decreased but remaining	0.40	stationary	167
8	F	50	DSM	0.22	2	bevacizumab × 1, ranibizumab × 1	0.22	regressed	0.22	regressed stationary	50
9	F	59	inferior staphyloma	0.30	3	aflibercept × 3	0.30	stationary	0.30	stationary	10
10	F	47	inferior staphyloma	0.52	1	bevacizumab × 1	0.70	stationary	0.70	increased	5
11	M	54	inferior staphyloma	0.40	1	bevacizumab × 1	0.40	stationary	0.40	regressed	38
12	F	55	inferior staphyloma	0.70	2	aflibercept × 2	0.70	decreased but remaining	0.10	increased	9

*yrs* years, *M* male, *F* female, *DSM* dome-shaped macula, *BCVA* best corrected visual acuity, *logMAR* logarithm of minimal angle resolution, *SRD* serous retinal detachment

## Discussion

In the current study, morphological features of DSM and inferior staphyloma with SRD were evaluated. SRD was associated with choroidal features such as thicker subfoveal thickness, greater variations in choroidal thickness, presence of abrupt changes in choroidal thickness and presence of large choroidal vessels. Axial length was shorter, subfoveal curve height was higher and inferior staphyloma was more frequent in eyes with SRD. Of the significant factors associated with SRD on univariate analysis, abrupt changes in choroidal thickness and shorter axial length were the two risk factors associated with SRD on multivariate analysis.

The pathophysiology of SRD in highly myopic eyes with DSM and inferior staphyloma is not clearly understood, as well as the pathophysiology of DSM and inferior staphyloma themselves. Previous studies have reported similarities between DSM and inferior staphyloma [17–19], and in one of the studies, greater change in choroidal thickness was a factor that may be involved in pathogenesis of SRD in patients with DSM and inferior staphyloma [19]. Recently, SRD of DSM and inferior staphyloma was explained by “choroidal funnel” hypothesis [20]. Pathogenesis of SRD in DSM and inferior staphyloma was explained by partial obstruction of choroidal blood flow which could be compared to a funnel. In the current study, abrupt change of choroidal thickness was more prevalent in eyes with inferior staphyloma (55.0%) compared to DSM (12.1%), and SRD was more common in eyes with inferior staphyloma.

The choroid is known the play an essential role in the pathophysiology of SRD in central serous chorioretinopathy (CSC), and fluorescein angiographic pattern of SRD in inferior staphyloma is similar to that of chronic idiopathic CSC [21]. CSC is a clinical entity of the pachychoroid spectrum disease, with choroidal vessel dilation, increased choroidal vascular hyperpermeability, choroidal inner layer thinning, and choriocapillaris ischemia playing an important role in the pathophysiology [22, 23]. Fluid accumulates in the subretinal space due to the oncotic pressure of the choroid combined with RPE dysfunction [24, 25]. Choroidal congestion can also be inferred from increased choroidal thickness. In the current study, subfoveal choroidal thickness was thicker in patients with SRD implying choroidal congestion as a potential underlying mechanism. In addition, greater variations in choroidal thickness, presence of abrupt changes in choroidal thickness and presence of large choroidal vessels may also imply focal choroidal congestion. The location of abrupt changes in choroidal thickness and large choroidal vessels were most commonly located within the SRD or at the edge, which also suggests their association with SRD. However, compared to CSC in which the choroidal congestion is more diffuse, choroidal congestion features in DSM/inferior staphyloma were found to be more focal and associated with the junctional area showing abrupt changes in scleral curvature. The presence of abrupt changes in choroidal thickness showed the highest odd ratio and remained a valid risk factor for SRD on multivariate analysis. Thicker subfoveal choroidal thickness, higher variation in choroidal thickness, and a higher incidence of large choroidal vessels may all be prerequisites for abrupt changes in choroidal thickness. This may also be due to the highly sensitive detection of abrupt choroidal thickness changes by three-dimensional reconstruction in a wider foveal area.

The other risk factor associated with SRD on multivariate analysis was shorter axial length, and axial length of eyes with SRD was shorter than that of eyes without SRD (25.55 ± 1.27 µm vs 29.67 ± 3.64 µm, *p* = 0.007). The axial length of inferior staphyloma was shorter than that of DSM (25.55 ± 0.90 mm vs 29.41 ± 2.93 mm, *p* < 0.001) which is in an agreement with previous studies. The axial length of eyes with horizontally oriented DSM (30.2 ± 1.81 mm) in the study by Garcia-Ben et al. was longer than that of eyes with inferior staphyloma (25.74 ± 1.42 mm) in the study by Lee et al. which is similar to the findings of this study [13, 26]. Also, shorter axial length may have resulted from measurement at the apex of the macular bulge, and in this study DSM/inferior staphyloma with SRD had a higher macular bulge than those without SRD. Another previously proposed pathophysiology of SRD is mechanical RPE damage from macular bulge leading to its dysfunction [5]. Higher bulge height may have led to more pronounced and prolonged RPE damage, as bulge height is known to gradually increase with time [27]. Focal scleral thickening under the macula is another EDI OCT finding in DSM patients [28]. Scleral thickening can cause obstruction of choroidal outflow, as seen in nanophthalmos, leading to subretinal fluid accumulation [29]. Although scleral thickness was not measured in the current study, shorter axial length in eyes with SRD could imply thicker sclera.

Although some researchers believe DSM to be a variant of interior staphyloma, they have distinct morphologic features in the macula area [30]. DSM is somewhat symmetrically concave from the fovea, whereas concavity is asymmetric in inferior staphyloma. The superior portion remains somewhat rigid, while the inferior portion expands out of the eye. In patients with SRD, inferior staphyloma was more common than DSM. This asymmetry may affect the choroidal vasculature, as abrupt changes in either scleral or retinal curvature at the macula may cause opposing forces, resulting in abrupt changes in choroidal vasculature between the two curvatures leading to SRD.

As for other complications other than SRD in highly myopic eyes with DSM/inferior staphyloma, retinoschisis was located in the extrafoveal region, which may be due to the macular bulge, as a macular buckle is used to treat severe retinoschisis by alleviating tractional forces at the fovea [31]. Although statistically not significant, some ocular comorbidities could be associated with presence of SRD. PED was more common in eyes with SRD. Deobhakta et al. also reported that PED often accompanies DSM with SRD and may suggest possible choroidal neovascularization [32]. ERM was more common in eyes without SRD, in which axial length was longer. Since posterior vitreous detachment, which is closely associated with ERM development, is expected to occur earlier in myopic eyes with longer axial length, this may explain the higher prevalence of ERM in this group [33].

In the treatment results of DSM/inferior staphyloma associated SRD with anti-VEGF agents, most SRD remained unchanged or even increased. SRD decreased during follow-up without treatment in 4 of 15 eyes. This suggests the lack of VEGF involvement in the pathophysiology of SRD associated with DSM/inferior staphyloma. Whether the fluid in SRD is exudative or transudative remains uncertain. RPE dysfunction due to choroidal congestion or mechanical damage from the macular bulge suggests the accumulation of exudative fluid. In contrast, abrupt changes in curvature leading to dissociation between layers, as seen in retinoschisis, and non-responsiveness to anti-VEGF agents are more indicative of transudative fluid. Fluorescein angiography of DSM/inferior staphyloma eyes does not show pooling of fluorescein, which is typically seen in other diseases showing SRD such as CSC; instead, variable hyperfluorescent signals are shown [13, 34]. Thus, no definite effective treatment modality has been suggested to date. Both photocoagulation and anti-VEGF treatment showed disappointing results in previous studies in SRD eyes with inferior staphyloma [35–37].

Limitations of this study include the retrospective and cross-sectional design of the study, hindering the investigation of dynamic changes in DSM/inferior staphyloma. Second, the inclusion of only DSM/inferior staphyloma and not all high myopia patients or patients with/without posterior staphyloma may limit investigation of incidence. Third, scleral thickness, whose hypothetical role in DSM has been proposed, was not measured due to the lack of adequate imaging modality. Also, the relatively small number of patients included requires validation of the results in prospective studies of a larger scale.

Despite the aforementioned limitations, this study investigated the pathophysiology of SRD in DSM/inferior staphyloma using multimodal imaging, suggesting that abrupt changes in curvature with macular bulging and shorter axial length result in focal choroidal congestion and possible RPE dysfunction overlying the junctional site, leading to SRD. Long-term follow-up studies are warranted to validate the results of this study, which will shed light on the pathophysiology of SRD development in DSM/inferior staphyloma.

## Supplementary Information

Below is the link to the electronic supplementary material.

ESM 1 (DOCX 856 KB)
