# Supplementary material for: Morphological characteristics of high myopes complicated by serous retinal detachment with dome-shaped macula or inferior staphyloma
Source: Graefes Arch Clin Exp Ophthalmol. 2025 Mar 25;263(7):1847–58. doi: 10.1007/s00417-025-06802-z (PMC12373529; doi:10.1007/s00417-025-06802-z)
Supplement: Supplementary file 1 — (DOCX 856 KB) [file 417_2025_6802_MOESM1_ESM.docx]

*Graefe's Archive for Clinical and Experimental Ophthalmology*

**Morphological Characteristics of High Myopes Complicated by Serous Retinal Detachment with Dome-shaped Macula or Inferior Staphyloma**

Jeong Hyun Lee^1,2^, Woosung Jeon^1,3^, Min Seok Kim^1,4^, Kwangsic Joo^1,4^, Se Joon Woo^1,4^, Joo Young Shin^1,2^, Jeeyun Ahn^1,2^

^1^Department of Ophthalmology, Seoul National University College of Medicine, Seoul, Korea

^2^Department of Ophthalmology, Seoul Metropolitan Government-Seoul National University Boramae Medical Center, Seoul, Korea

^3^Department of Ophthalmology, Seoul National University Hospital, Seoul, Korea

^4^Department of Ophthalmology, Seoul National University Bundang Hospital, Seongnam, Korea

**Co-correspondence to:**

Jeeyun Ahn, MD, PhD

Department of Ophthalmology, Seoul National University College of Medicine, Seoul Metropolitan Government- Seoul National University Boramae Medical Center

20, Boramae-ro 5-gil, Dongjak-gu, Seoul, Korea, 07061

Tel: +82-2-870-2416, Fax: +82-2-831-0714

Email: autre24@gmail.com

Joo Young Shin, MD, PhD

Department of Ophthalmology, Seoul National University College of Medicine, Seoul Metropolitan Government-Seoul National University Boramae Medical Center

20, Boramae-ro 5-gil, Dongjak-gu, Seoul, Korea, 07061

Tel: +82-2-870-2414, Fax: +82-2-831-0714

Email: joo0shin@gmail.com

**
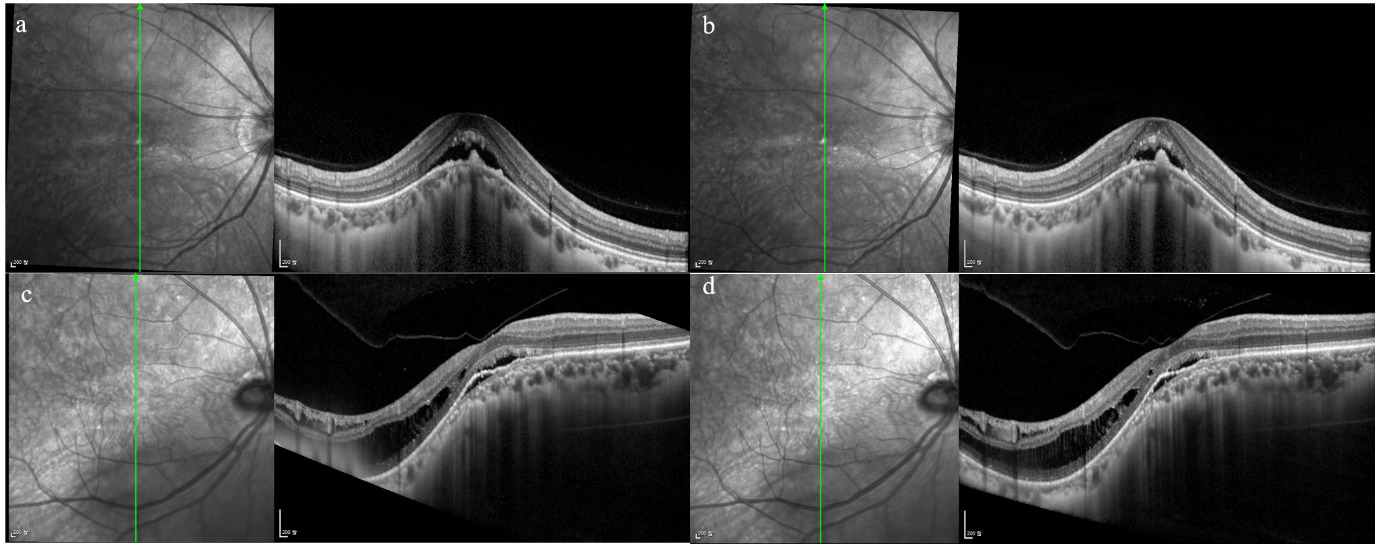
Supplementary Fig. 1** Representative OCT images of patients with dome-shaped macula (Supplementary Fig. 1a, Supplementary Fig. 1b) and inferior staphyloma (Supplementary Fig. 1c, Supplementary Fig. 1d) who showed response after intravitreal bevacizumab injection (Supplementary Fig. 1b, Suppliemntary Fig. 1d)
